# A Digital Network Approach to Infer Sex Behavior in Emerging HIV Epidemics

**DOI:** 10.1371/journal.pone.0101416

**Published:** 2014-07-03

**Authors:** Abhinav Kapur, John A. Schneider, Daniel Heard, Sayan Mukherjee, Phil Schumm, Ganesh Oruganti, Edward O. Laumann

**Affiliations:** 1 Pritzker School of Medicine, University of Chicago, Chicago, Illinois, United States of America; 2 Department of Medicine, University of Chicago, Chicago, Illinois, United States of America; 3 Department of Health Studies, University of Chicago, Illinois, Chicago, United States of America; 4 Department of Statistical Science, Duke University, Durham, North Carolina, United States of America; 5 Public Health Management Institute, SHARE-India, Hyderabad, Andhra Pradesh, India; 6 Department of Sociology, University of Chicago, Chicago, Illinois, United States of America; Public Health Agency of Barcelona, Spain

## Abstract

**Purpose:**

Improve the ability to infer sex behaviors more accurately using network data.

**Methods:**

A hybrid network analytic approach was utilized to integrate: (1) the plurality of reports from others tied to individual(s) of interest; and (2) structural features of the network generated from those ties. Network data was generated from digitally extracted cell-phone contact lists of a purposeful sample of 241 high-risk men in India. These data were integrated with interview responses to describe the corresponding individuals in the contact lists and the ties between them. HIV serostatus was collected for each respondent and served as an internal validation of the model’s predictions of sex behavior.

**Results:**

We found that network-based model predictions of sex behavior and self-reported sex behavior had limited correlation (54% agreement). Additionally, when respondent sex behaviors were re-classified to network model predictions from self-reported data, there was a 30.7% decrease in HIV seroprevalence among groups of men with lower risk behavior, which is consistent with HIV transmission biology.

**Conclusion:**

Combining the relative completeness and objectivity of digital network data with the substantive details of classical interview and HIV biomarker data permitted new analyses and insights into the accuracy of self-reported sex behavior.

## Introduction

Since the first cases of HIV were reported among high risk men in 1981, global HIV prevention research has been dominated by individual-level assessments that utilize self-reported behavior to determine risk of HIV acquisition. Methodologies used in collecting self-reported risk behavior such as computer assisted surveys or online health diaries have contributed to improving the accuracy of sensitive sex behavior data [1], [2]. However, the continued disconnect between these subjectively-reliant methods and objective clinical outcomes has been well established as a major impediment to accurate interpretation of HIV study findings [3], a feature described by some as the “behavior biology conundrum” [4]. Self-reported sex behavior is error-prone because of the sensitive nature of this behavior as well as well as the complicating effects of partner and contextual variation [5]. Fundamentally, self-reported sex behavior may be insufficient to determine actual sex behavior in addition to the downstream HIV infection risks that occur. In contrast to individual level self-reported information, a network based approach that elicits information from other network members may help improve inference of sex behavior [6].

To date, researchers have mostly utilized the reports of one network member on a second and suggested that such dyadic reports reflect more upon the rater’s behavior than the network member of interest [7], [8]. Some of these studies have continued to prioritize self-reported risk behavior as an accurate assessment of actual behavior yet when empirically examined they have been found to lack validation. Other studies on sex behavior have also compared self-report to the report of others in the context of timing of sexual encounters [9], or whether two partners agree on the existence of a sex tie between them [10]. These have found significant incongruity across reports of shared behavior such as sex tie as well as across types of reporters.

Newer social network research that leverages existing digital communication networks such as emails [11], or cell phone calls [12], [13], could help us infer behaviors more accurately. These data generate networks without the biases involved in collecting the names of network members. They also produce large networks rapidly without the problems of matching names or other personal attribute information. Moreover, the scale of networks generated could be useful for the plurality of reports on the sex behaviors of individuals within the network. The plurality of reports offered by new digital network data provides opportunities to include multiple raters instead of one, and allows for raters to be weighted by their relationship to the individual of interest. The plurality of reports clearly can strengthen sex behavior inference [6], and the consensus structures developed through social network analysis might be useful in obtaining accurate information [14]. Improvements in plurality of reports on behavior inference could be made even stronger if large numbers of reporters were objectively identified and recall bias in name generation was limited [15]. In fact, recall bias becomes a particularly important problem when sex partners are elicited [5].

Accurate inference of sex behavior can be further strengthened by linking distinct HIV transmission risks to specific behaviors. In terms of both HIV transmission and acquisition, for example, vaginal sex presents a much lower risk compared to anal sex [16], [17]. Populations such as men who have sex with men (MSM) were the first to acquire HIV in the United States (primarily through anal intercourse) and MSM continue to have the highest rates of HIV transmission in emerging epidemics internationally. Yet even among MSM, important HIV transmission rates vary depending upon specific sex behaviors or positions. For example, an estimated 20% increased HIV transmission potential in receptive over insertive anal sex [17], represents a risk difference critical to biomedical HIV prevention research. Given this known risk difference between insertive and receptive anal sex, HIV serostatus information could further help us better assess the accuracy of sex behavior information.

This study compares network-generated sex behavior to self-reported data by combining linked cell phone contact list data with respondent interviews. Interviews of respondents included self-reported behavior and the reported behavior of others in contact lists, many who were themselves respondents. The study then compares both sets of data to HIV serostatus to determine the approach that most accurately reflects sex behavior using a mixed-effects model [18], [19]. Combining the relative completeness and objectivity of digital network data with the substantive details of classical interview data and biomarker results permitted new analyses and insights into the accuracy of self-reported sex behavior.

## Materials and Methods

### Setting and Study Population

The setting for this study was in a large city in Southern India. The study took place at a constellation of 20 well characterized social venues- “cruising areas” -where MSM congregate to socialize and where paid and unpaid sex is common. The study population included the following: individuals identifying as male between 18–39 years of age who visit one of the 20 venues, report anal/oral intercourse with another man within the previous 12 months, own and are in possession of at least one cell-phone at the time of recruitment, speak English or one of two local languages, and were able and willing to provide written informed consent for study participation. Protocols were approved by institutional review boards at the University of Chicago and SHARE-India.

### Respondent Recruitment

**Figure 1:**
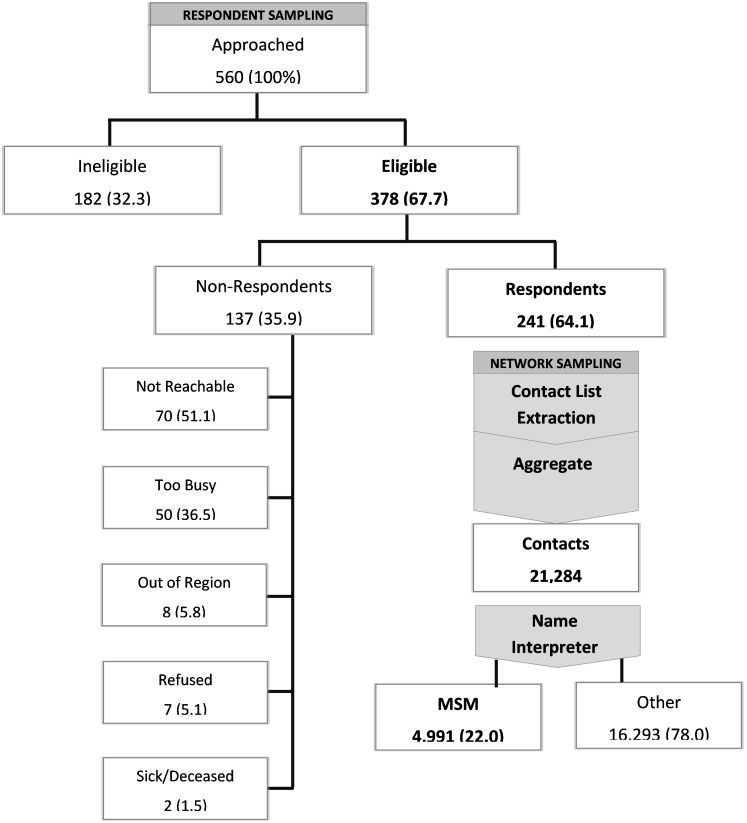
Sample recruitment schema of study respondents (n = 241), Southern India 2010. Non-respondents were eligible participants who did not present for informed consent at a nearby field office following field recruitment. Name interpreters are a series of questions asked about contact list members of respondents. In this case respondents identified contact list members as MSM or not MSM.

Time Location Cluster Sampling (TLCS) was conducted [20], [21]. Previously a sampling frame was established including 20 separate venues and 3 hour periods where MSM can be recruited. This covered public venues such as railway stations, theatres, small restaurants, parks, museum grounds etc. with MSM on a given night ranging from 30–200 at each site. Venues with smaller numbers of MSM (<30), such as massage parlors and private residences were not included for logistical and cost-efficiency reasons. In concert with two local partnering non-governmental organizations, we re-identified all major venues frequented by MSM and the days of the week and times of day when MSM frequent the venues. Every month we randomly selected (without replacement) 15 venues from the sampling frame and then randomly selected one of the 3 hour periods associated with the venue. The two-member data collection team approached MSM at the venue and evaluated inclusion criteria as described above. Men who approached the team for enrollment or who enrolled previously (verified by cell phone number) were ineligible. We recorded limited demographic data on men who refused participation and counted all men passing through. A schematic for recruitment of the study sample can be found in **Figure 1**.

**Figure 2:**
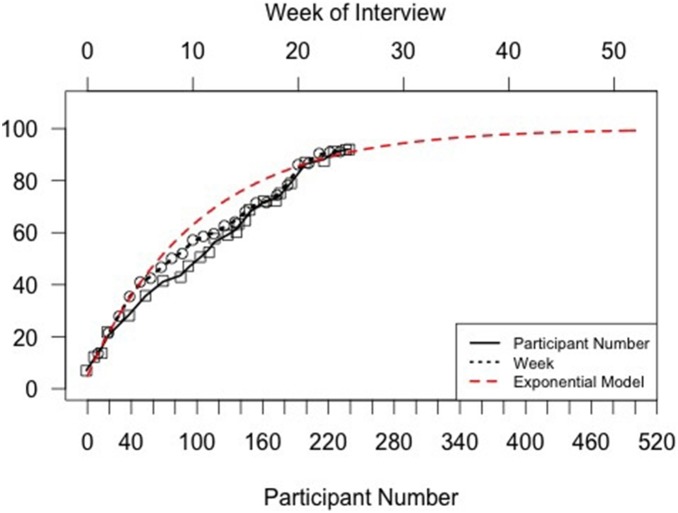
Network redundancy curve of study respondents used to determine adequate sample size for network model (n = 241). Curve fit from data on index of respondents and week of respondent interviews versus network size to exponential model. The data were fit to a scaled/shifted exponential cumulative distribution function f(x) = 99.2–95.9e∧(−4.9x) where x represents the index of the respondent and f(x) represents network size. Data approach horizontal asymptote at approximately 240 respondents.

In order to determine when network saturation was achieved, we followed recruitment through a redundancy curve (**Figure 2**). This demonstrated that to achieve network saturation in this region (where each subsequent recruit is >95% likely to already have been linked in the network through another participant’s contact list), we required a sample size of 245. Once this was achieved over a six month period, any further recruitment was stopped.

### Data Measures

**Figure 3:**
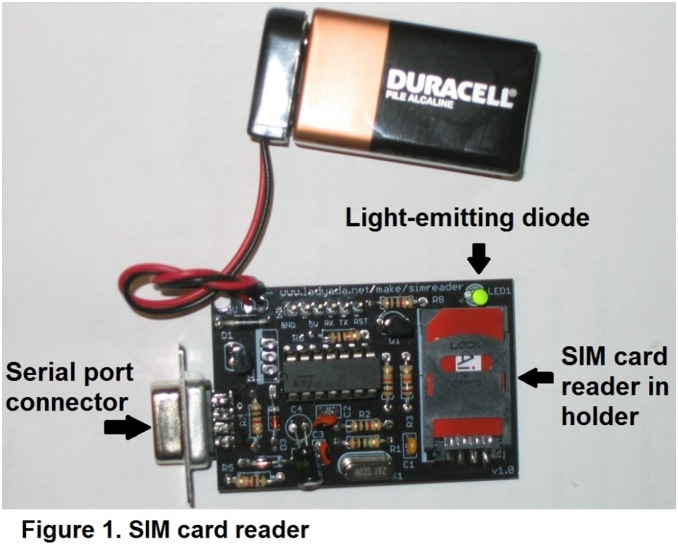
Subscriber Identity Module (SIM) card reader. The SIM card reader [22], was assembled using a kit from Adafruit Industries (New York, NY). The card reader is operated by means of pySIM [23], a free open-source SIM card-reading software package.

To overcome interviewer and respondent burden from classic name generators that require recall and cataloguing names in a roster, the use of a SIM card reader was adopted (**Figure 3**). SIM cards are utilized in nearly all cell-phones outside of the United States. The SIM card reader was assembled using a kit from Adafruit Industries [22], and operated by means of pySIM [23], a free open-source SIM card-reading software package. The software is written in Python and modified for compatibility with the SIM card reader. The software allows extraction of phone book entries which include phone numbers, name information and the SIM card serial number from each respondent’s cell phone. For this study, phonebook entries were extracted and sent directly to a file for respondent interview. Call frequency and call duration are not collected as part of the SIM card reader. The network was generated by matching phone numbers across contact lists. Betweeness centrality [24], and bridging as measured by a link-deletion approach [25], were calculated for all MSM in the network.

The following individual and relational characteristics were collected from respondents about themselves and about each MSM network member in their cell phone contact list. Variables were based upon those in previous work with this population [26]–[28]: caste (caste system is an Indian social class hierarchy system), religion, marital status, sexual position (mostly insertive, mostly receptive, versatile), and any previous sex work history. Sex position was collected from respondents about their perception of their network members’ typical sex positions (regardless of whether they are a sex partner or not), as well as for the predominant sex position used with sex network members. Tie measurements also included the duration of relationship and frequency of communication with each network member. While we did not include call frequency in the SIM card reading which could reflect closeness of a tie, one item from Morrison’s closeness scale [29], adapted to this setting [30], was collected for each contact to describe tie closeness (very close, a little close, not close). Cell phone specific information was also collected: number of handsets/SIM cards per respondent, duration of SIM card possession, whether handsets/SIM cards are shared, and previous SIM card numbers. In order to address potential limitations of incomplete network data obtained, we also collected information on social and sexual network members who may not utilize cell phones and network members who may utilize cell phones but may not be in respondents’ contact lists.

All study respondents provided dry blood spots for HIV testing and three sequential antibody tests (Vironostika HIV Uni-Form II Ag/Ab, bioMérieux; Tridot, Biomed Industries; Retrocheck, Qualpro Diagnosistics) were conducted using methods described previously [31], and in accordance with National AIDS Control Organization guidelines [32]. HIV test results and referrals were provided to study participants following locally developed procedures [33].

### Model Generation and Analytic Plan

The network-based mixed effect model predictions of MSM sex position incorporate the reports of other network members on the individual of interest and structural network features such centrality and bridging [25], [34]. The key point of the modeling in this study is to predict behavior of individuals based on tie information and structural network features. This allows for quantitative behavioral estimates that we contrast with self-report. The model predicts attributes of network members by a respondent given the network members’ other attributes. The attributes considered in the model were religion, type of MSM, marital status, receiving money for sex, meeting at a sexual hotspot, and caste. The predictions are then used to compare with self-report and determine variation in agreement as a function of network type and attribute. Analysis of this type for determining behavior and relationships in sexual networks based on multiple reports has been done in several instances, including Helleringer et al. [10], and Adams and Moody [6]. Brewer et al. [5], examined dyad characteristics to determine if particular individual or pair attributes were consistent with concordant information. The main statistical tool used in our predictive modeling was a mixed effect model [18], [19] of the form.
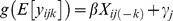
where 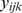
corresponds to the k^th^ attribute of alter *i* as reported by ego *j*, *X_ij(–k)_* are the set of all attributes of alter *i* except the k^th^ attribute as reported by ego *j*, 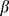 are coefficients modeling effects across all respondents, *γ_j_* represents the random effect specific to respondent *j*, and *g* represents a link function. For binary attributes we used a logit link. Sex behavior had multiple categories corresponding to a multinomial link. We used leave-one-out procedures to test model fits and the basic assumptions of the mixed effects model. We also applied a block model [35], to this data which showed latent structure suggesting individuals block memberships formed according to marital status. This model was applied to the entire network as well as sex and social sub-networks. It also incorporated structural features of the network such as centrality and bridging [25], [34], as covariates.

Comparing self-reported attributes of 241 respondents to the model predictions was of central importance in the analysis. This provided a comparison of one’s self-reported identification (i.e. sex position) versus the perception of this identification by others and a quantitative prediction of this perception given the network data. For sex position, we used a logistic mixed effects model, where individuals were classified as either being insertive or receptive. Because MSM in this context can also be versatile, engaging in both insertive and receptive sex, we used a thresholding approach. The threshold approach is. is an extension of Krackhardt’s work on Consensus Structures [14] which we used to transform ego-alter predictions from the model for a particular alter to a sex position assignment for that alter as well as to allow for individuals to be classified as versatile. We defined the following quantity.
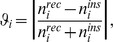
where 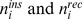 are the number of insertive and receptive predictions for alter i. A value of 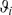 equal to 1 means there is complete concordance in the predictions based on reports in the data, and the individual is classified strictly as insertive or receptive. A value of 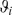 equal to 0 means that an individual has equal numbers of insertive and receptive predictions. If 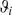 is smaller than a threshold 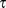 then the alter is classified as versatile; otherwise the alter is classified by the majority prediction. Values of 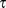
close to 0 result in only individuals with nearly equal numbers of insertive and receptive predictions to be classified as versatile with the remaining individuals being classified as either strictly insertive or receptive, whereas values near 1 result in nearly all individuals being classified as versatile. We used a threshold of 0.5, which corresponded to a ratio of 3∶1 or smaller comparing the MSM type predicted more often to that predicted less often. This gave a distribution of MSM sex roles consistent with what we expect to see based on the literature. This approach also allowed us to address model uncertainty resulting from differing ego reports on a given alter. We obtained similar results using an approach wherein we assigned prior probabilities of each MSM sex role to individuals and then, assigned MSM sex roles to minimize loss according to our loss function.

### Ethical Considerations

All procedures were approved by Institutional Ethics Committees in the United States and India. As is typical of social network analysis, we collected limited information on third parties that are provided by consented study respondents [36], [37]. The key concerns to study participants in the pure academic context are 1) lack of consent on the part of persons named by respondents, and 2) the possibility of identifying individuals by combining collateral information [37], [38]. We utilized a secure data management system that included password protected computers where data were entered and encrypted file transfer to a secure server. All data at site of collection were destroyed and analytic data subsequently protected by a Federal Certificate of Confidentiality. Network visualizations and study results were presented internally and to local community partners to receive feedback prior to public release. All figures presented in this manuscript were anonymized prior to internal review and were found to protect personal information.

## Results

**Figure 4:**
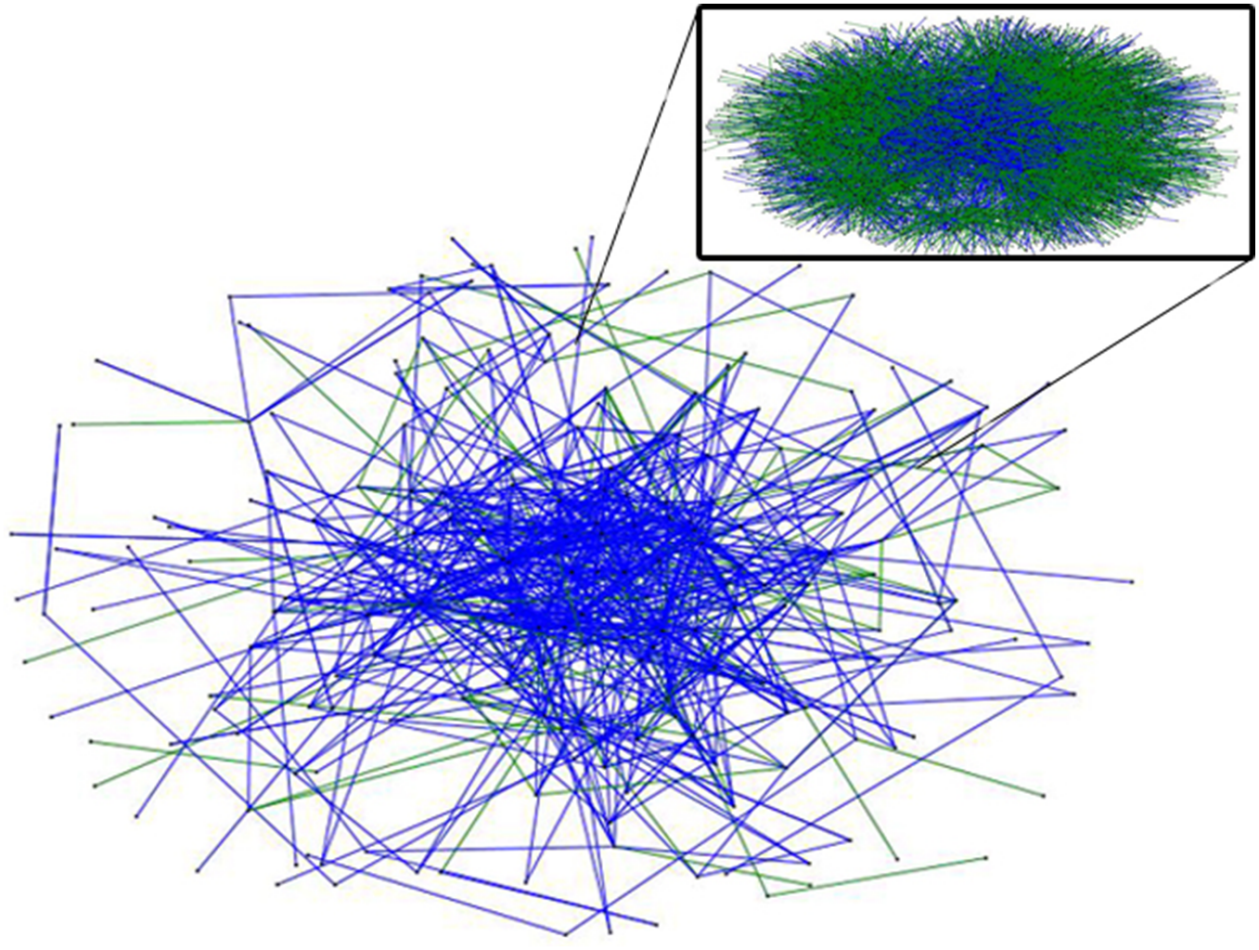
Digital communication network of MSM respondents (n = 241; 706 ties). Ties are designated by blue for social and green for sex. Inset network is of the augmented network which includes MSM respondents and all MSM from respondent cell phone contact lists (n = 4991; 6458 ties). Network matches were determined but utilizing cell phone numbers as identifiers.

MSM study respondents were recruited until the chance of a new respondent already being part of the network was >95%. All respondents were in the contact list of at least one other respondent. This process resulted in a network of MSM (n = 241; 706 ties) and an augmented MSM network including those who were not interviewed directly (n = 4991 MSM; 6548 ties) (**Figure 4**). The augmented cell-phone network included the 241 respondents and all other MSM within their cell-phone contact lists. Both *social* and *sex* sub-networks of the cell-phone network were also analyzed separately. The network-based model predictions of sex behavior incorporate the reports of other network members on the individual of interest as well as structural features of network members such as centrality and bridging [25], [34].

### Network Based Model Predictions versus Self-Reported Data

**Figure 5:**
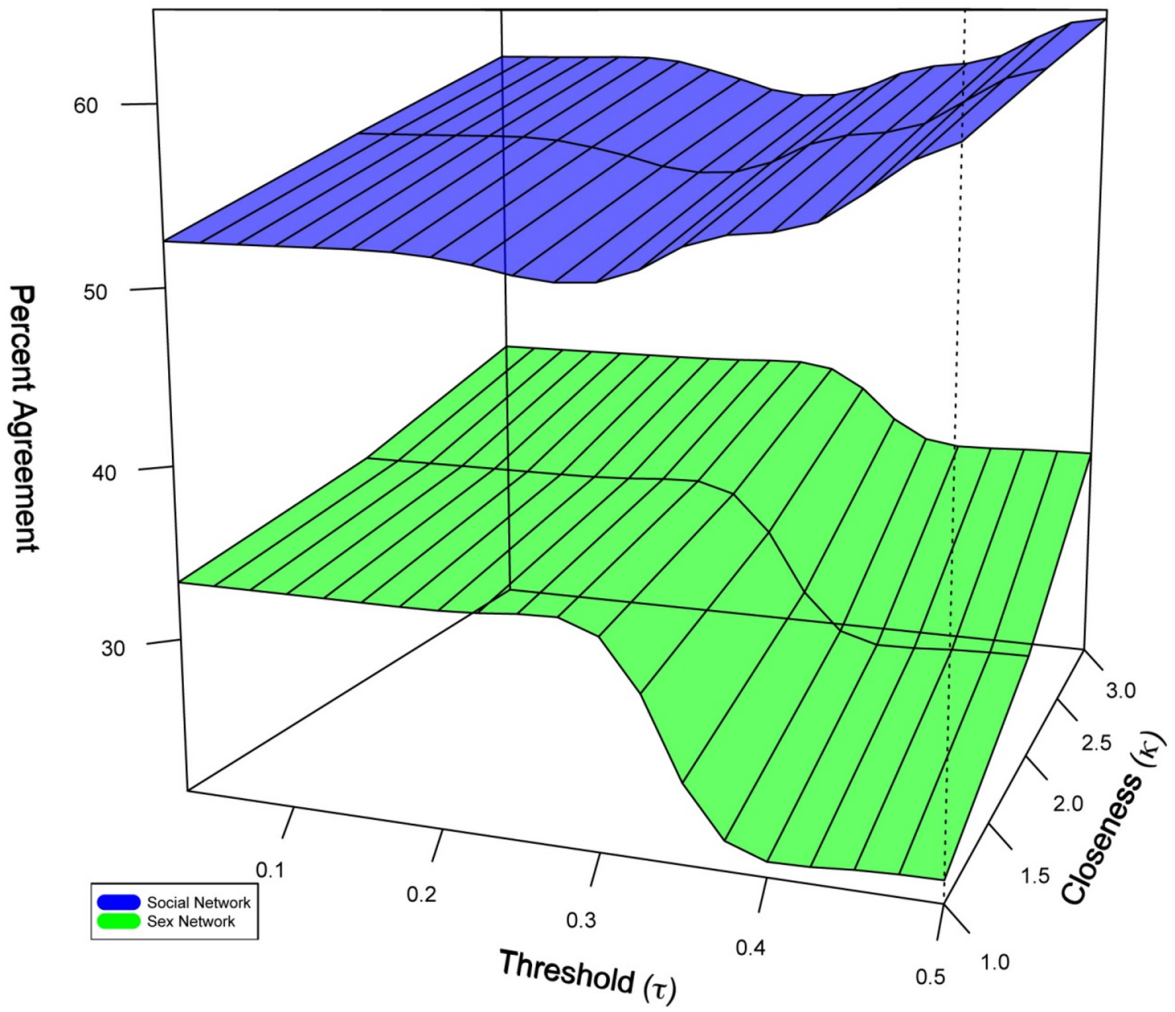
Percentage of MSM sex behavior agreement with self-report in social compared to sex networks. More agreement between self-report and model predictions is evident in the social network (upper surface) than the sex network (lower surface); however agreement between self-report and model predictions increases across thresholds as closeness (κ) between sex network members increases. (τ) serves as a metric comparing proportions of model predictions for insertive and receptive sex positions. (κ) serves as a measure of closeness indicated by score from 1.0 (least close) to 3.0 (closest).

Our primary results indicate that network-based model predictions of sex position and self-reported sex position had limited correlation (54% agreement). Further, there was variability in this agreement based upon network type: the agreement between self-report and model predictions within the sex network was 36% and agreement in the social network was 61% (See **Figure 5**). This finding suggests that sex behavior inference based upon self-reports may be inaccurate due to discordance between sex behavior roles as observed in sex venues by MSM and the sex behavior of the same MSM as reported by sex partners. **Figure 5** also demonstrates that tie closeness (κ) increased the agreement between self-report and the network model, a finding that was most apparent in the sex network where the average agreement across all thresholds increased from 28.6% for ties classified as ‘somewhat close’ or ‘not close’ to 38.1% for ties classified as ‘very close’.

**Figure 6:**
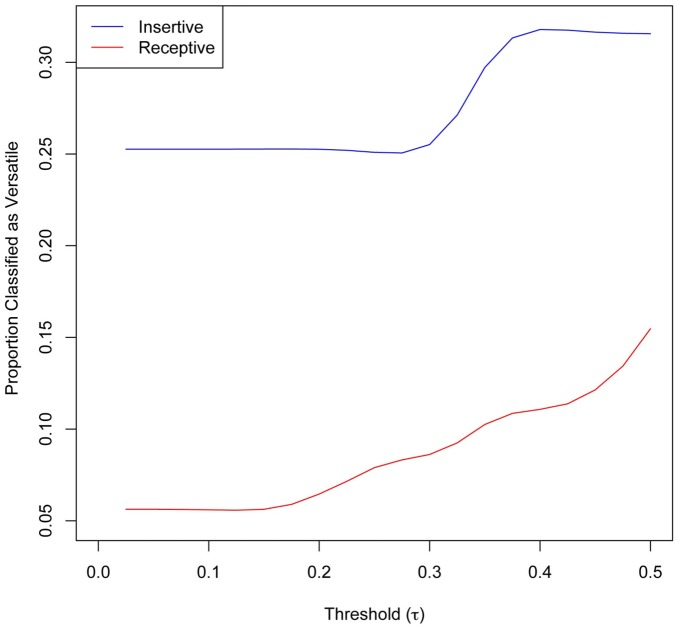
Self-reported insertive only and receptive only sex behavior classified as versatile according to the network model. Versatile sex means engaging in both insertive and receptive anal sex. Respondents self-reporting insertive sex only were more likely to be classified as versatile by the network model. (τ) serves as a metric comparing proportions of insertive and receptive model predictions.

Not only did self-report behavior differ from model predictions by *type* of network, but how it was classified differed depending on the type of behavior reported: versatile behavior (both insertive and receptive) in the sex network was misclassified by self-report as insertive (See **Figure 6**). This is an expected finding in sex venues where insertive MSM often also take on a receptive role as sex workers – making them versatile in their *actual* sex behavior. Agreement between model predictions and self-report was much higher for other identifiable attributes in this context such as religion (data not shown). Attributes such as religious affiliation are more likely to be comparable between self-report data and network predictions because they tend to be explicit in this context and less dynamic than sex behavior.

### Biomarker Validation of Network Inferred Sex Behavior

**Figure 7:**
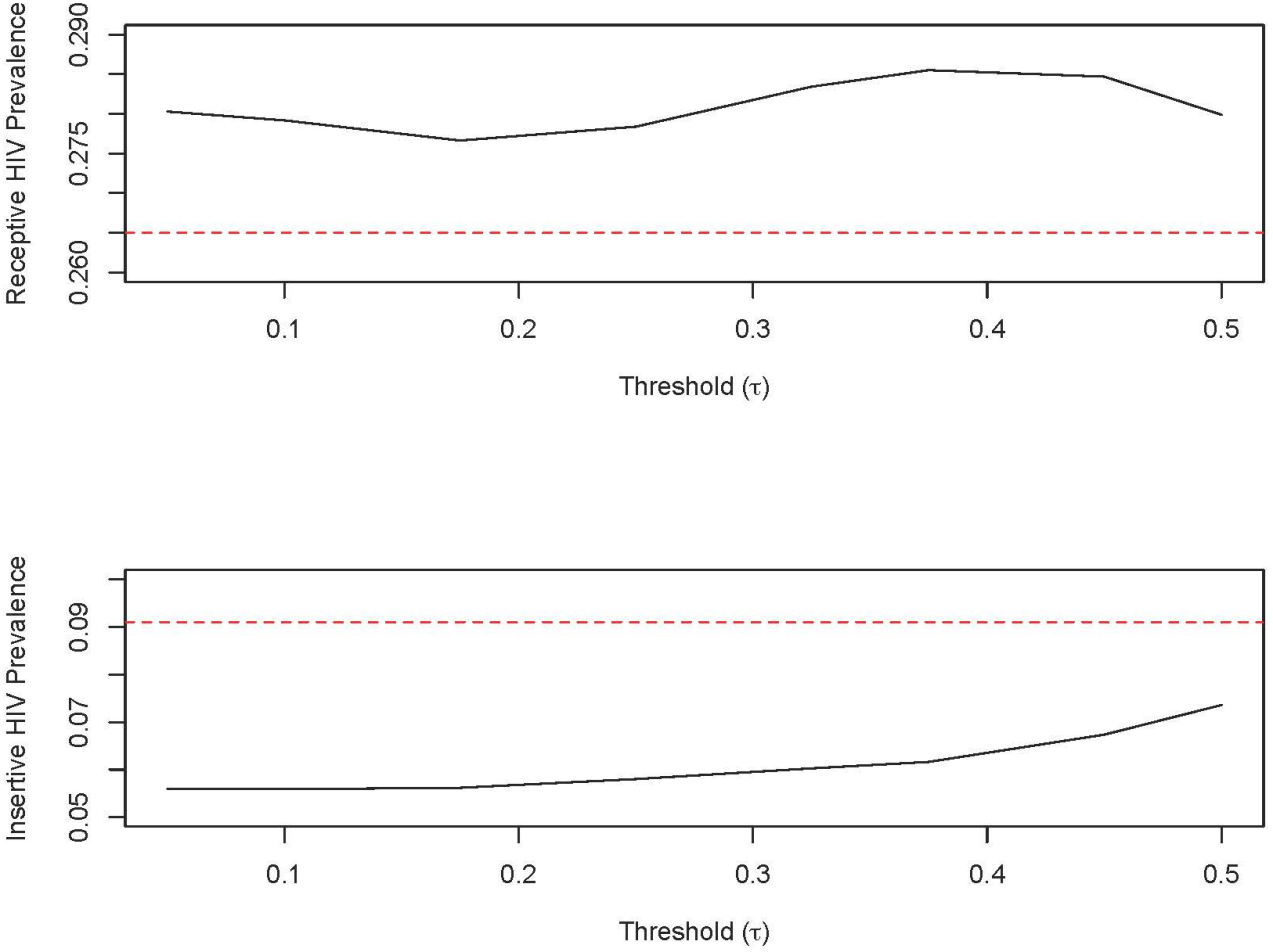
HIV seroprevalence of MSM according to network predictions (dark line) of sex roles and self-report of sex role (red). The network predictions (black line) reveal higher HIV seroprevalence among receptive MSM and lower HIV seroprevalence among insertive MSM as compared to self-reported sex positions (dotted red line). These higher and lower HIV seroprevalence rates designated by network predictions match the direction in which biologic HIV transmission differences would confer HIV seroprevalence among receptive (black line is higher) and insertive (black line is lower) MSM.

The overall HIV seroprevalence rate in this population was high at 23.4%. Seroprevalence rates have been shown to vary by self-reported risk behavior with receptive MSM in this setting having the highest rates of infection followed by versatile (both insertive and receptive) and then insertive [39], [40]. This pattern is consistent with biologic HIV transmission risk for each sex behavior with insertive sex having the least chance of HIV acquisition among MSM [17]. The network predictions revealed higher HIV seroprevalence among receptive MSM and lower HIV prevalence among insertive MSM across thresholds of role designations (**Figure 7**). These higher and lower HIV prevalence rates designated by network predictions match the direction in which biologic HIV transmission differences would confer HIV seroprevalence among receptive (higher) and insertive (lower) MSM. Thus, re-classification of MSM sex behavior according to the network model was consistent with biologic HIV transmission risk as insertive MSM in the network model had consistently lower rates of HIV seroprevalence across all thresholds when compared to self-report. Additionally, when respondent sex behaviors were re-classified from self-report to network model predictions, there was a 30.7% decrease in HIV seroprevalence in the insertive group which is consistent with the decreased HIV transmission risk of this sex behavior among insertive MSM.

## Discussion

Our network model represents a shift in how sexual behavior can be inferred. Significantly, it shifts the current reliance on self-reported data by incorporating sex behavior reports from a plurality of network members and using structural features of networks to predict sex behaviors.

Validating whether the network model predictions improve upon self-report is difficult due to the challenge in measuring sex behavior objectively. Self-reported behavior is often not highly associated with HIV infection, an observation that may be due to social desirability bias, measurement error, sexual network characteristics (which can increase risk independently of individual behavior) [2], as well the possibility that people have safe sex with risky partners and risky sex with safe partners [41]. Previous attempts at measuring other stigmatized behavior using network data such as substance use among adolescents have been limited because they lacked a validation standard (such as a toxicology assessment) and because the network was mostly limited to dyads (two individuals). To address these limitations, our model incorporated the plurality of network reports on a given individual. Network reports and each individual’s network position resulted in a sex behavior classification for each individual. This new sex behavior classification was then compared against each individual’s HIV serostatus. The new classification was found to be more congruent with what would be expected from biologic transmission related to specific sex behaviors as compared to behaviors reported by individuals about themselves.

The reason for modest congruence between the network model and self-reported sex behavior is likely driven by the potential response bias secondary to social desirability of self-reported behaviors. However, it may also be driven by dissonance between a sexual identity typical in the Indian context [42], of fixed sex-behavior roles that may be incongruous by the objective observations of sex behaviors by network members. For example, one may consider himself as the insertive partner and assume such an identity, however, when in sex-venues where transactional sex occurs, participate as a receptive partner. One would then expect that these individuals would subsequently have higher rates of HIV as compared to individuals whose self-identity matched behavior; which is what we found in our biomarker validation.

The approach discussed in this paper offers an important step in moving beyond the monolith of self-report data which form the current foundation of most HIV prevention research. This conventional approach can lead to potential systematic underestimation of sex behavior in cases of heterogeneous risk potential (such as the MSM case), which in turn can limit the interpretation and implementation of HIV research results. By removing these limitations, the new approach might, for example, allow for emerging biomedical interventions to be distributed more efficaciously to individuals more at risk, such as self-described “insertive” MSM who also assume receptive sex positions. A digitally-derived network model that leverages hybrid network analytic approaches improves inference of sex behavior in emerging global HIV epidemics. The increasing availability of digital communication technology among international populations could allow for greater inference capacity as well as dissemination of future HIV interventions.
